# Early clinical and radiological outcomes of the new porous titanium shell in combination with locking screw in revision total hip arthroplasty

**DOI:** 10.1186/s42836-023-00177-4

**Published:** 2023-05-08

**Authors:** Shahril R. Shaarani, Monketh Jaibaji, Khaled M. Yaghmour, Georges Vles, Fares S. Haddad, Sujith Konan

**Affiliations:** 1 Department of Trauma & Orthopaedics, University College London Hospital, London, NW1 2BU UK; 2 University College London, London, NW1 2BU UK

**Keywords:** Revision arthroplasty, Revision acetabular shell, Bone loss, Paprosky

## Abstract

**Introduction:**

Extensive acetabular bone loss and poor bone quality are two key challenges often encountered in revision total hip arthroplasty. A new 3D-printed porous acetabular shell has been made available with the option to insert multiple variable-angle locking screws. We sought to evaluate the early clinical and radiological outcomes of this construct.

**Methods:**

A retrospective review of patients operated by two surgeons was performed in a single institution. Fifty-nine revision hip arthroplasties were performed in 55 patients (34 female; mean age 68.8 ± 12.3 years) for Paprosky defects I (*n* = 21), IIA/B (*n* = 22), IIC (*n* = 9), III (*n* = 7) between February 2018 and January 2022 using the novel porous titanium acetabular shell and multiple variable angle locking screws. Postoperative clinical and radiographic outcomes were locally maintained. Patient-reported outcome measures collected included the Western Ontario and McMaster Universities Osteoarthritis Index (WOMAC), the Oxford Hip Score, and the 12-item Short Form Survey.

**Results:**

After a mean follow-up of 25.7 ± 13.9 months, two cases of shell migration were noted. One patient had a failed constrained mechanism and received revision to a cemented dual mobility liner. No other acetabular shells showed any evidence of radiographic loosening at the final follow-up. Preoperatively, 21 defects were classified as Paprosky grade I, 19 grade IIA, 3 grade IIB, 9 IIC, 4 grade IIIA, and 3 IIIB. The mean postoperative WOMAC function score was 84 (SD 17), WOMAC (stiffness) 83 (SD 15), WOMAC (pain) 85 (SD 15), and WOMAC (global) 85 (SD 17). The mean postoperative OHS was 83 (SD 15), and mean SF-12 physical score was 44 (SD 11).

**Conclusion:**

The additional augmentation of porous metal acetabular shells with multiple variable-angle locking screws provides reliable initial fixation with good clinical and radiological outcomes in the short term. Further studies are needed to establish the medium- and long-term outcomes.

**Level of evidence:**

IV.

## Introduction

Extensive bone loss and poor bone quality are two key challenges often encountered in revision total hip arthroplasty (THA) [1]. The goal of acetabular reconstruction is to achieve a long-lasting fixation, re-establish the center of rotation of the hip and provide a stable joint in a manner that deals minimal harm to the patient [2]. Porous metal acetabular shells have been proven to be a reliable option for reconstruction, demonstrating excellent long-term survivorship [3, 4]. The durable highly-porous surface allows for the ingrowth of host bone and remodelling at the bone-implant interface, ensuring a stable fixation [5]. In vitro analysis has shown similar results for locking screws used in combination with an acetabular shell, with less micromotion at the bone-implant interface and enhanced osteointegration [6]. As this implant has only recently become available, there is a paucity of clinical data on this highly porous titanium acetabular shell with locking screws. We therefore sought to investigate its early clinical and radiological outcomes. We hypothesized that this novel acetabular revision component would achieve excellent survivorship and osseointegration, regardless of indications for revision and degree of acetabular bone loss.

## Methods

### Design

A retrospective analysis of collected data was carried out in a single institution between 2 orthopedic surgeons. No ethical approval was required from our institutional review board.

### Patient population

Between February 2018 and January 2022, fifty-five consecutive patients who underwent revision THA for the failure of the acetabular component were included in the study. No other implants were used during this period. Baseline characteristics such as age, gender, American Society of Anaesthesiologists (ASA) grade and BMI were noted.

### Implant

The REDAPT shell (Smith and Nephew, London, UK) is composed of a titanium alloy (Conceloc™) with porosity ranging between 60–80% and pore sizes of 200–900 µm. A coefficient of friction of 0.95 was reported. The implant has a 9-hole (48–58 mm diameter) and a 12-hole option (60–80 mm diameter). The screw holes accept a 6.5-mm cancellous threaded screw (range 15–50 mm in length) with a variable angle range of up to 12º. A standard cemented XLPE liner can be used in all cup sizes. A dual mobility liner can be used in shell sizes equal to or greater than 54 mm.

### Operative technique

All procedures were performed by two senior orthopedic surgeons. Preoperative planning of shell sizes was performed with digital templating, and with primary component sizes known. All patients were operated on in lateral decubitus position after receiving intravenous tranexamic acid and antibiotics (teicoplanin and gentamicin) at induction. Previous incisions were utilized if possible, and a posterior approach was used in all cases and the incision was extended when necessary. A minimum of 5 samples was routinely sent for microbiological analysis for infected cases. Following the removal of the acetabular component, cement and membrane, sequential acetabular reaming was carried out until the desired size for the press-fit was reached. When necessary, an augment was used to deal with essential non-contained defects. When bone stock was compromised the non-modular shell was placed where the bone is present and the liner was cemented in the correct orientation. Modular shells might be used in type 1 and 2 Paprosky defects.

A trial shell was Inserted to verify the appropriate size and orientation with the transverse acetabular ligament. Next, the selected shell was impacted into the acetabulum and stability was again assessed at this point. The fixation was then augmented using locking screws via the pre-drilled holes in the shell, with the kickstand screw in the ischium. Regular screws could be used first to compress the shell to the bone before the application of the locking screw. Our preferred technique, when press fit can be achieved, is for the use of locking screws. Once adequate fixation was established, the screw holes were filled with hole covers to prevent cement from reaching the screw heads or from going behind the shell. If the XLPE liner was used, this was cemented into the shell using polymethylmethacrylate (PMMA). Occasionally, a smaller size dual mobility liner (cemented Polar dual mobility (Smith & Nephew Inc.)) was used with reverse augmentation technique [7] and placed eccentrically to allow for better reconstruction of the center of rotation. Bone loss from the anterior column of the acetabulum was ignored.

The femoral stem was assessed intraoperatively. The Redapt revision femoral stem (Smith & Nephew, Memphis, TN, USA) was used for aseptic loosening of the femoral prosthesis, periprosthetic fracture, and conversion of DHS and infected cases. The fixed femoral stems were left in situ with a Bioball adaptor. Primary THR stems were used in the cases of revision hip resurfacing with either uncemented Polar (Smith & Nephew, Memphis, TN, USA) or cemented Exeter stems.

Immediately after the operation, patients with Paprosky IIC defects or above were advised to engage in toe-touch weight bearing. If the defect size was less than IIC then partial weight bearing was permitted. At 6 weeks, all patients were mobilized (full weight bearing) with or without crutches. No crutches were used beyond 3 months.

### Clinical outcome measures

Patients were reviewed postoperatively against radiographs at 6 weeks, 6 months, 12 months and annually. Clinical assessment was conducted using standardized patient-reported outcome measures (PROMS), which included the WOMAC score, OHS, and SF-12.

### Radiological outcome measures

**Fig. 1:**
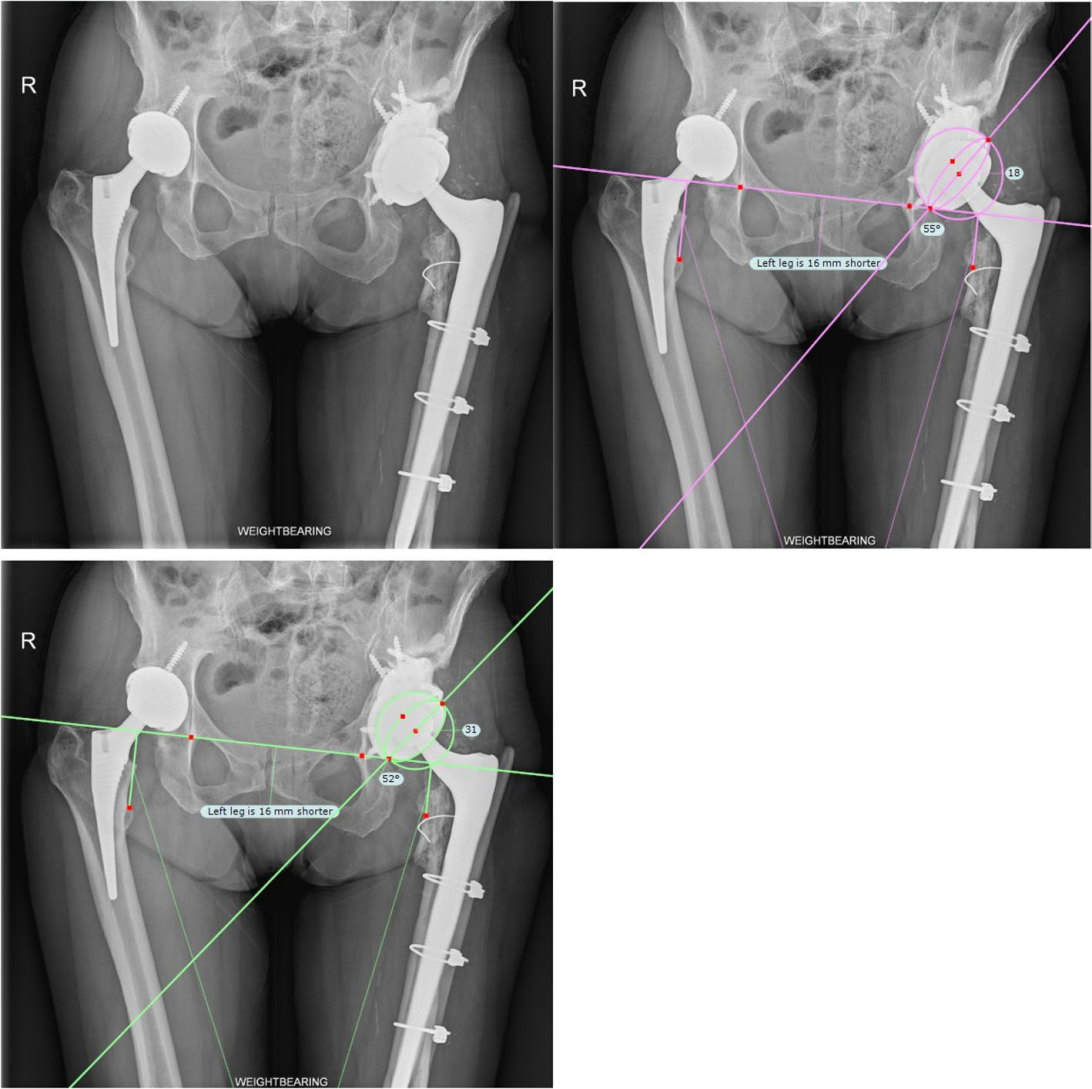
Measurement of the Redapt/Polar abduction and anteversion angle using TraumaCad

The acetabular defects were graded and classified using the preoperative X-ray according to the Paprosky classification [8]. Postoperative radiographs were evaluated by two orthopedic surgeons independently for any signs of loosening, migration, or any other complications using TraumaCad® (BrainLab, Chicago, IL, USA) (Fig. 1). The native tools of TraumaCad® were used to measure acetabular inclination and acetabular anteversion. The built-in “Cup version and LLD analysis” tool of the software automatically calculated the angles once the tools were properly positioned by the user on the acetabular cup and the pelvis. The inclination is also referred to as the abduction angle. LLD was measured using the trochanteric method. In terms of wear assessment, digital tools provided with the TraumaCad™ software, such as a ruler, bevel protractor and template of concentric circles, were used. By using these tools, the distance between the superior outer surface of the acetabular cup and the superior border of the femoral head at 90º of the femoral head’s horizontal axis was measured. The difference between the postoperative AP radiograph and the final follow-up X-ray was taken as wear. All measurements were done three times with the average logged.

### Statistical analysis

Descriptive statistics were calculated for clinical factors (age, ASA grade, BMI and follow-up). Student *t*-tests were used for comparing continuous variables, paired *t*-tests were used for paired variables and χ^2^ test was used for categorical variables. The Spearman-rho correlation was identified between variables. *P* < 0.05 were considered significant. All statistical analyses were carried out by using SPSS Version 27.

## Results

### Procedures performed

The patient demographics are shown in Table 1. The mean follow-up lasted for 25.7 months (SD 13.8, range 4–52). The indications for revision were aseptic loosening (*n* = 21), prosthetic joint infection (*n* = 11), peri-prosthetic fracture (*n* = 8), recurrent dislocation (*n* = 3), failed hip fracture fixation with acetabular damage (*n* = 3), tumour metastasis (*n* = 2), hip resurfacing revision with/without pseudotumour (*n* = 5), squeaking ceramic-on-ceramic articulation (*n* = 1), native hip dislocation (*n* = 1), cemented prosthetic stem fracture with aseptic loosening (*n* = 1), neck of femur fracture with the arthritic hip (*n* = 1), acetabular fracture (*n* = 1) and acetabular erosion from previous hemiarthroplasty (*n* = 1). All the failed neck of femur fracture fixations (*n* = 3) were previously treated with dynamic hip screws. With regards to the patients with periprosthetic fractures, there was one case of a Vancouver C fracture around Birmingham’s hip resurfacing arthroplasty, and six cases of Vancouver B2 fractures around a femoral stem, where acetabular erosion was noted secondary to reduced shell anteversion. One periprosthetic fracture was due to acetabular fractures (Table 2).

**Table 1:** Patient demographics

Patient characteristics	Value	SD
Mean age (years)	68.8	12.3
M:F	25:34	
Mean BMI (kg/m^2^)	26.6	5.9
Mean F/u (months)	25.7	13.7
ASA grade		
II	32	
III	19	
IV	1

**Table 2:** Indications for revision and classification of bony defects

	Number of revisions
**Characteristics**	
Aseptic loosening	21
Prosthetic joint infection	11
Recurrent dislocation	3
Failed dynamic hip screw fixation of previous neck of femur fracture	3
Failed Birmingham hip resurfacing ± pseudotumour	5
Metastatic disease	2
Acetabular erosion from previous hemiarthroplasty	1
Squeaking ceramic-on-ceramic articulation	1
Native hip joint dislocation	1
Broken cemented stem + acetabular osteolysis	1
Neck of femur fracture with arthritic hip	1
Peri-prosthetic fracture	8
Vancouver B2	6
Vancouver C	1
Acetabular fractures	1
**Paprosky classification **	
I	21
IIA	19
IIB	3
IIC	9
IIIA	4
IIIB	3

Paprosky I defect was seen in 21 patients, 19 had Paprosky IIA, 3 had an IIB defect, 9 patients had IIC, 4 had IIIA defects and 3 patients had an IIIB defect.

**Fig. 2:**
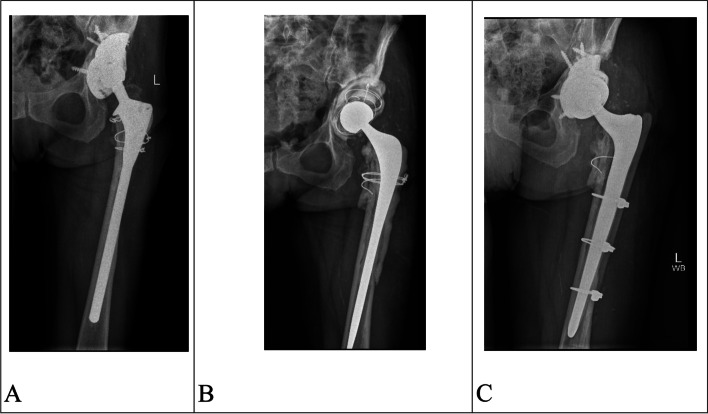
**A** Revision THA with septic loosening of a custom-made shell with extensive femoral and acetabular bone loss. **B** Situation after the first stage where a temporary spacer was made from a long Exeter stem and 2 Exeter cups using the first-generation cementing technique. **C** Situation after the second stage showing a fully porous shell and augment with locking screws in all three pelvic bones, a cemented dual mobility liner and a monolith tapered fluted revision stem

The acetabulum alone was revised in 6 cases with the acetabulum and femur being revised in 38 cases. Femoral revisions were performed using monolithic tapered fluted stems (Redapt stem; Smith & Nephew, London, UK) [9, 10] or with cement-in-cement polished taper stems (Exeter V40 125 mm stem, Stryker Orthopaedics, Weston, FL, USA) [11]. Seven femurs were implanted with a primary uncemented prosthesis (Polar stem, Smith & Nephew, Baar, Switzerland). The median number of screws utilized was 4 in the Redapt acetabular component, with a mode cup size of 54 mm. Screws were drilled through the augments in all six cases where augments were required (Fig. 2). A dual mobility bearing (Polar cup dual mobility, Smith & Nephew, London, UK) was used in 29 cases. A 20-degree lipped liner was used in the remaining cases. A mode Polar cup size of 43 mm was employed as part of the dual mobility construct.

### Clinical outcomes

There were three clinical failures, two shell migrations, and one revision of the liner (Table 3). No revision of a shell has been undertaken to date.

**Table 3:** Three failures in the case series and their clinical outcomes

**Case 1:** A 77-year-old female with a Paprosky 3A defect experienced migration of the shell. She initially presented with a shell failure following acetabular revision for eccentric liner wear. The Redapt shell with locking screws was not used at the index revision. A second revision was planned to use the new porous shell but, unfortunately, augments were not available on the day and a jumbo shell was used with five locking screws for stability. The shell showed early migration but then stabilized and as the patient was pain-free, she opted not to have any further surgery.
**Case 2:** A 44-year-old female had multiple surgeries in another country for developmental dysplasia of the hip (DDH) with a query Chiari osteotomy, and eventually had a THR at the age of 32. The primary hip had a high hip centre, and this eventually failed with failure of the primary shell and pelvic discontinuity. The patient had a Paprosky 3A defect and had no issues until 34 months postoperatively when repeat radiograph showed medialization of the shell. Currently, there are plans for a revision surgery, but this requires planning with a custom-made implant over the next coming months.
**Case 3:** An 82-year-old female with a Paprosky 2C defect originally underwent a staged revision for prosthetic joint infection (PJI) (McPherson IIIC2). She underwent revision of articulation from lipped liner to polar dual mobility liner without revision of the porous revision shell and has remained stable at 45-months postoperatively.

Two patients died 6 months post-surgery due to unrelated causes. Three other patients deceased between 1 to 2 years following revision surgery.

Mean PROM scores at final follow-up: WOMAC function score was 84 (SD 17), WOMAC (stiffness) 83 (SD 15), WOMAC (pain) 85 (SD 15), WOMAC (global) 85 (SD 17). The mean OHS was 83 (SD 15), the mean SF-12 physical score was 44 (SD 11), mean postoperative SF-12 mental score was 56 (SD 10).

### Radiological outcomes

**Fig. 3:**
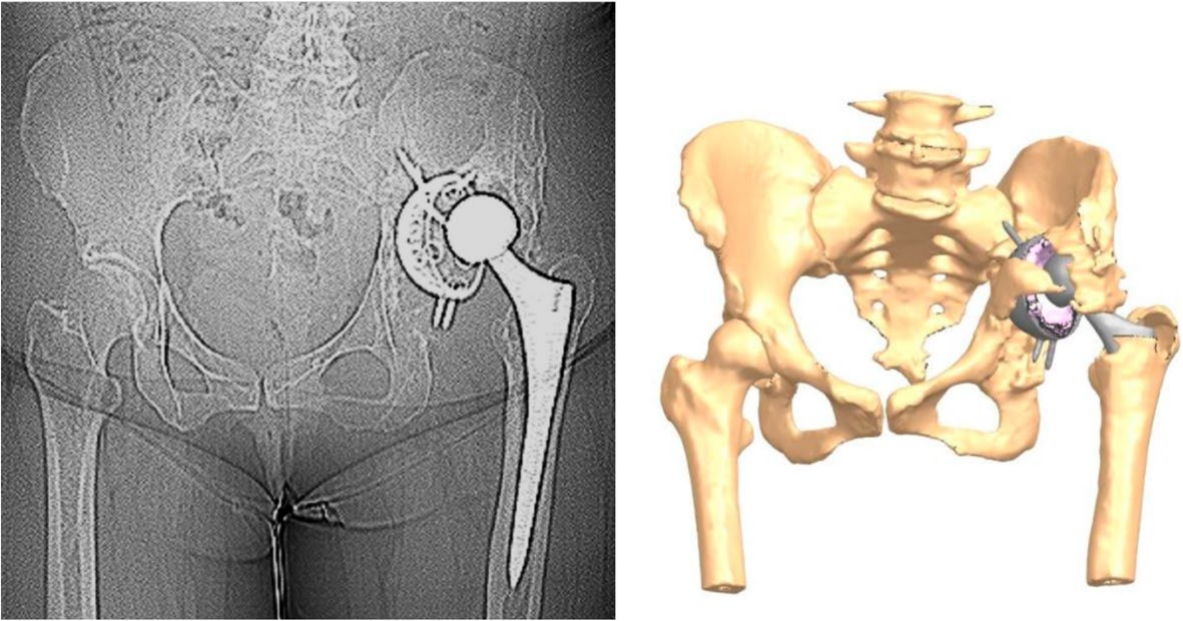
Patient with medialization and proximalization of the cup as described in Table 3, Case 2

Radiographs were available for all patients. Two cases developed shell migration as aforementioned (Fig. 3). No radiographs demonstrated radiolucency on any of the postoperative radiographs. One non-revised implant with cup migration showed good signs of osteointegration (Table 3, case 1). The mean Redapt shell abduction angle was 45.3 degrees (SD 7.1), Redapt anteversion angle 22.5 degrees (SD 9.2), Polar cup abduction angle 43.9 degrees (SD 8.1), and Polar cup anteversion angle 25 degrees (SD 8.6). The mean leg length discrepancy was -3.45 mm (SD 11).

### Statistical analysis

The significant correlation between independent variables is shown in Table 4.

**Table 4:** Spearman correlations between independent variables in the study

Variables	Spearman correlation	2-tailed significance
Sex—Age At Surgery	0.17	0.93
Sex—ASA	-0.011	0.946
Age At Surgery—screws	0.05	0.973
Age At Surgery—redapt version	-0.013	0.931
Weight (kg)—ASA	-0.035	0.838
Weight (kg)—redapt version	-0.038	0.826
Weight (kg)—augment	-0.036	0.835
BMI—screws	0.045	0.799
BMI—redapt cup size	-0.032	0.855
BMI—polar cup version	0.005	0.985
Paprosky—screws	0.017	0.907
Paprosky—redapt cup size	-0.008	0.958
Paprosky—Limb length discrepency (LLD)	0.002	0.990
Paprosky—polar cup angle	0.045	0.825
Redapt cup size—augment	-0.005	0.973
Polar cup size—redapt version	-0.015	0.941
Limb length discrepency (LLD)—augment	0.042	0.788
Redapt angle – augment	0.029	0.853

## Discussion

This is the first case series in a single institution to report on the clinical and radiological outcomes of this novel implant. We reported good short-term results up to this point, with no shell revisions in our first 55 patients. We acknowledge the limitations of our study in terms of sample size and length of follow-up, however, this is to be expected, due to the relative infancy of the implant.

Several studies have demonstrated excellent long-term outcomes of porous acetabular shells, with survivorship between 75–96% [12, 13]. Prieto et al. [14] demonstrated 94% survivorship at 5 years when revision due to acetabular component failure was used as an endpoint. Another study on porous acetabular shells by Löchel et al. [4] demonstrated a 92.5% acetabular shell revision-free survivorship at 10 years, with their revision rate for aseptic loosening being 5.6%. Two out of their three cases of aseptic loosening had no screw fixation as initial stability was deemed acceptable. Consequently, it was advised that screw augmentation should be used in all cases, a process that this porous revision shell with locking screws can facilitate. Unfortunately, at this stage, a direct comparison between these studies and ours is not yet possible. This combination of highly porous titanium shells with pre-drilled variable angle locking screw holes is one of several options on the market for the reconstruction of challenging acetabular defects [15].

The use of 3D printing to manufacture the revision shell allows for more screw holes in total and the screw holes to be placed closer to the periphery of the shell, therefore allowing for easier access to the ischium and pubis. The use of these so-called ‘kickstand screws’ [16] prevents abduction failure of the shell, whereas placement of screws in the dome alone may increase micromotion at the ischial rim [17, 18]. Using screws in this configuration provides greater interface stiffness, allowing the construct to resist hip vector forces in the superior and lateral direction when walking [19].

The various methods of revision hip arthroplasty with impaction bone grafting (IBG) and acetabular component cementation, custom-made tri-flange cups and ring/cage construct have their disadvantages. IBG’s lack of a biological fixation poses a notable disadvantage [20], carries a risk of graft resorption and infection [21] and is highly dependent on a meticulous surgical technique [22]. Custom-made tri-flange cups require the usage of a three-dimensional CT scan to generate a 3D acrylic model of the affected pelvis. The results of several studies do raise concerns regarding an unacceptably high dislocation rate [23, 24], and could be a result of excessive soft tissue dissection leading to superior gluteal nerve denervation during the process of flange placement [25]. The lag time of at least six weeks from ordering the custom component to performing the surgery could lead to possible further bone loss, resulting in the construct not fitting the acetabular defect as desired [2, 12, 15]. The initial ring and cage construct had mid-term results showing survivorship standing between 70–80% [26, 27]. This was improved with the cup-cage modification, which shows a survivorship of 85–97% at 2 to 5 years with good patient-reported outcomes [28–30]. The numbers included in these studies were small and long-term data on this implant configuration are not yet available. There remains at least a theoretical concern about cage fracture over time [31]. These concerns make a strong case for the use of porous shells and augments in some cases of pelvic discontinuity [4].

Pelvic discontinuity is a unique challenge in revision hip arthroplasty. Our approach involves the use of a pelvic distraction in a technique similar to that described by Sporer et al. [32]. In their study, elastic recoil of the soft and fibrous tissues across the discontinuity site aided in the initial stability of the shell-screw construct. This approach effectively allows the shell to be used as a hemispherical base for osteosynthesis, which can enhance osteointegration of the acetabular component. We also used the shell itself as a distraction device by impacting an oversized shell into the acetabulum. This contrasts with the original distraction technique where pins were connected to the ilium and ischium via a universal distraction device [22]. This can have the advantage of shortening the operative time as well as being a technique much more familiar to the arthroplasty surgeon.

Uncemented extra-large or ‘jumbo’ shells are a technically simple option that can be used in high-grade defects and some studies have demonstrated low complication rates [15]. A key advantage is the reduced need for bone grafting owing to their ability to maximize surface contact with the acetabulum [15]. The implant does raise the hip center of rotation, which may affect functional outcomes [13]. Oblong shells have also been tried with Paprosky III defects in several studies with follow-up ranging from 84–108 months [33, 34]. Unlike ‘jumbo’ shells, they do not raise the hip center of rotation, but they have yet to be evaluated in significant numbers.

In this case series, there was a trend for an increased number of screws used and increased anteversion in the Redapt shell with age. There was a negative correlation between weight and ASA grade, Redapt shell anteversion and the use of augments. Interestingly there was a positive correlation between body mass index (BMI) and the number of screws, Redapt cup size and the Polar cup anteversion. With increasing Paprosky grades, there was a positive correlation between limb length discrepancy and the Polar cup abduction angle, but a negative correlation between limb length discrepancy and the Redapt cup size. There is a negative correlation between the Redapt cup size and the use of augments; and the Polar cup size and the Redapt cup anteversion. The use of augments was associated with increasing limb length discrepancy (LLD) and Redapt cup angle.

Hence, our data showed that age and BMI affected the severity of acetabular bone loss and might increase the number of screws and the Redapt cup size. The usage of augments assisted in reducing the size of the Redapt cup but could affect abduction angle.

The limitations of the study include the small sample size, as this is a new implant used in our institution. Ultimately, the small population size of each cohort limits the power of the data analysis. Additionally, radiographic follow-up lasted only for 2 years. Thirdly, the retrospective nature of the research leaves the data confounded. This leads to a lower level of evidence and limits the definitive conclusions.

## Conclusion

Porous acetabular shells have well-established favorable long-term outcomes. The additional augmentation with variable angle locking screws, in the short term, provides reliable fixation with good clinical and radiological outcomes. The implant is also technically user-friendly. We have shown, in our case series, that there is a 5% complication risk for all indications for revision THA. While these early results are promising, further studies are needed to provide long-term data. In addition to a longer clinical and radiological follow-up of the patients in this study, a radiostereometric analysis study is currently underway at our institution to provide more information on its initial stability. In conclusion, this implant may be considered a viable option in the majority of acetabular revisions either with a lipped liner or a reverse augmentation cementing technique.

## Data Availability

Not applicable.
